# Generation and physiological roles of linear ubiquitin chains

**DOI:** 10.1186/1741-7007-10-23

**Published:** 2012-03-15

**Authors:** Henning Walczak, Kazuhiro Iwai, Ivan Dikic

**Affiliations:** 1 Tumour Immunology Unit, Department of Medicine, Imperial College London, 10N5 Commonwealth Building, Du Cane Road, London W12 0NN, UK; 2 Department of Biophysics and Biochemistry, Graduate School of Medicine and Cell Biology and Metabolism Group, Graduate School of Frontier Biosciences, Osaka University 2-2 Yamada-oka, Suita, Osaka 565-0871, Japan; 3 Institute of Biochemistry II, Medical Faculty of the Goethe University, University Hospital Building 75, Theodor-Stern-Kai 7, 60528 Frankfurt am Main, Germany

## Abstract

Ubiquitination now ranks with phosphorylation as one of the best-studied post-translational modifications of proteins with broad regulatory roles across all of biology. Ubiquitination usually involves the addition of ubiquitin chains to target protein molecules, and these may be of eight different types, seven of which involve the linkage of one of the seven internal lysine (K) residues in one ubiquitin molecule to the carboxy-terminal diglycine of the next. In the eighth, the so-called linear ubiquitin chains, the linkage is between the amino-terminal amino group of methionine on a ubiquitin that is conjugated with a target protein and the carboxy-terminal carboxy group of the incoming ubiquitin. Physiological roles are well established for K48-linked chains, which are essential for signaling proteasomal degradation of proteins, and for K63-linked chains, which play a part in recruitment of DNA repair enzymes, cell signaling and endocytosis. We focus here on linear ubiquitin chains, how they are assembled, and how three different avenues of research have indicated physiological roles for linear ubiquitination in innate and adaptive immunity and suppression of inflammation.

## 

Ubiquitination was first recognized for its function in tagging proteins for destruction by the proteasome [1-5], but is now known to be one of the major types of post-translational modifications necessary for proper functioning of signaling cascades [6-8]. The attachment of ubiquitin molecules to their targets occurs through reactions mediated by proteins of three classes, acting in sequence: a ubiquitin-activating enzyme, E1, which contains an active-site cysteine to which the carboxy-terminal glycine of ubiquitin becomes attached through a reactive thioester bond; a ubiquitin-conjugating enzyme, the E2, to which the ubiquitin is transferred by an analogous reaction; and a ubiquitin ligase, the E3, which catalyzes the attachment of the ubiquitin to a lysine in the target protein [4,5,9-11]. Seven of the 76 amino acids of ubiquitin are lysines, which can themselves be targeted by ubiquitination to generate polyubiquitin chains of different linkage types depending on which lysine residue acts as the acceptor site for the incoming ubiquitin [12-14]. In an exception to this pattern, linear ubiquitin chains can be generated by the formation of a peptide bond between the carboxy-terminal glycine of the incoming and the amino-terminal methionine residue of the preceding ubiquitin molecule [15]. Recent research has established the identity and composition of an E3 ubiquitin ligase that generates linear ubiquitin chains, and has shown that these chains play an important part in several innate and adaptive immune signaling pathways, including the one triggered by tumor necrosis factor (TNF) [16-21]. Here we review what is known about the process by which linear ubiquitin chains are assembled, and how they contribute to TNF receptor 1 (TNFR1) signaling.

## LUBAC and the assembly of linear ubiquitin chains

The assembly of linear ubiquitin chains is unusual in three ways. First, as we have already mentioned, the linkage does not involve any of the lysine residues in the ubiquitin molecule, but occurs between the amino-terminal methionine of one ubiquitin and the carboxy-terminal glycine of the next in the chain. For this reason, linear ubiquitin chains are also known as M1-linked chains. The second unusual feature of linear ubiquitin chain assembly is that it is the E3 that determines the nature of the linkage in these chains [15] - a decision that is normally the prerogative of the E2, at least in reactions involving RING-class E3s [22]. The linear ubiquitin chain E3 is now known to be composed of three proteins. The first two of these - the heme-oxidized IRP2 ubiquitin ligase-1 (HOIL-1, also known as HOIL-1L and RBCK1) and the HOIL-1-interacting protein (HOIP, also known as RNF31) - were identified as part of this multi-component E3 by Kirisako *et al. *[15], who also coined the term linear ubiquitin chain assembly complex (LUBAC) for this novel type of E3.

**Figure 1:**
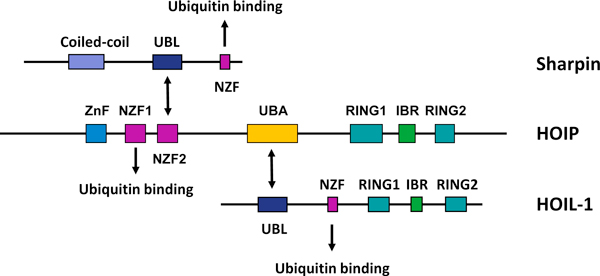
**Schematic representation of the LUBAC components, SHARPIN, HOIP and HOIL-1**. There is significant sequence homology (45% identity) between the carboxyl terminus of SHARPIN and the amino terminus of HOIL-1, each of which contains a UBL and an NZF motif. HOIP is the catalytic subunit of the tripartite LUBAC with SHARPIN and HOIL-1 as accessory factors that bind via their respective UBL domains to the NZF2 and UBA domains of HOIP, respectively. HOIP, SHARPIN and HOIL-1 also bind to ubiquitin chains through NZF-mediated interactions. The functions of the ZnF domain of HOIP and the coiled-coil domain of SHARPIN are currently unknown. The RBR domain of HOIP, but not of HOIL-1, is responsible for linear ubiquitin chain generation by LUBAC. Arrows indicate confirmed interactions between the proteins. Abbreviations: ZnF, zinc finger; NZF, Npl4 zinc finger; UBL, ubiquitin-like domain; UBA, ubiquitin-associated domain; IBR, in-between RING domain; RBR, RING-IBR-RING domain.

Subsequent research, however, revealed that LUBAC also contains a third component, SHARPIN (SHANK-associated RH domain interacting protein), whose carboxy-terminal region has high sequence similarity with the amino-terminal part of HOIL-1 [19-21]. The structural features of the three components of LUBAC and their interactions are schematically illustrated in Figure 1. All three contain ubiquitin-binding domains whereby they may bind to ubiquitin or to one another through ubiquitin-like (UBL) domains. HOIP is the central architectural component of the tripartite LUBAC, binding to both HOIL-1 and SHARPIN through their respective UBL domains. The stoichiometry of the three components that make up the 600 kDa LUBAC is currently unknown and it is also possible that complexes consisting of only two of the three factors exist [15]. In addition, it appears that in different cell types varying amounts of HOIL-1, HOIP and SHARPIN are present independently of the other LUBAC components. It is therefore possible that these proteins may also serve functions that are independent of LUBAC activity [19-21].

Several lines of evidence indicate that LUBAC generates exclusively linear ubiquitin chains: (i) LUBAC can generate ubiquitin chains with lysine-less (K0) ubiquitin *in vitro *[15,18,21]; (ii) LUBAC is unable to generate ubiquitin chains from amino-terminally tagged ubiquitin [15,19]; and (iii) mass spectrometric analysis of polyubiquitin chains generated *in vitro *by LUBAC reveals linear ubiquitin linkages [15].

## Where is the ubiquitin ligase activity of LUBAC and how is it activated?

There are two classes of E3s: RING (really interesting new gene) or U-box-type E3s catalyze the E2-mediated transfer of ubiquitin to target proteins [23,24], whereas in the case of HECT (homologous with E6-associated protein C-terminus)-type E3s ubiquitin is first transferred to the E3 by the formation of a thioester bond, and then from the E3 to the substrate. Both HOIL-1 and HOIP contain a RING-in-between-RING (IBR)-RING (RBR) domain (Figure 1), and hence form part of the RBR subclass of RING-E3s, so in principle either HOIL-1 or HOIP could account for the ubiquitin ligase activity of LUBAC. However, the combination of recombinant SHARPIN and HOIL-1 cannot generate linear ubiquitin chains *in vitro*, whereas recombinant HOIP together with HOIL-1 or SHARPIN (or of course both) can; moreover, overexpression of these combinations is also capable of activating NF-κB, one of the key transcription factors activated by TNF (see below) [19-21].

This is in line with experiments showing that, despite the fact that HOIL-1 and HOIP both contain an RBR domain (Figure 1), it is the RBR of HOIP that mediates the formation of the linear ubiquitin linkage in these different complexes because the intact RBR of HOIP, but not of HOIL-1, is required for LUBAC activity [15]. Indeed, despite its containing an apparently complete RBR domain [25,26], no linear ubiquitination activity has so far been detected for recombinant wild-type HOIL-1 in ubiquitination assays *in vitro*. It is possible, however, that interactions with partners other than HOIP and SHARPIN, or perhaps post-translational modification, may induce its activation.

If HOIP is the active E3 in LUBAC, what is the contribution of HOIL-1 and SHARPIN? The answer to this question and to the question of HOIL-1 E3 activity may lie in a mechanism recently reported for Parkin, another RBR-containing E3, which closely resembles HOIL-1 in domain structure [27,28]. Parkin is auto-inhibited by its UBL and this auto-inhibition may be relieved by binding to a co-factor or a substrate [29]. The zinc finger and the UBL domains of HOIL-1 and SHARPIN are crucial for activation of the linear-ubiquitin-generating activity of HOIP [16], and it may be that the binding of SHARPIN and/or HOIL-1 to HOIP relieves an auto-inhibition in HOIP in a way that is analogous to the activation of Parkin by binding to a partner (K Rittinger and B Stieglitz, personal communication). No qualitative differences have yet been discovered in the potential of SHARPIN and HOIL-1 to unleash the linear-ubiquitin-generating capacity of HOIP, although they seem likely to exist. It is tempting to speculate that SHARPIN and HOIL-1 may direct the linear ubiquitination activity of HOIP to different targets.

It remains to be determined whether there are binding partners for HOIL-1 other than HOIP and SHARPIN, and, if so, whether this results in HOIL-1-mediated generation of linear or other ubiquitin chain linkages. Recent results from Rachel Klevit and colleagues on Parkin and another RBR-domain-containing protein, human homologue of Ariadne (HHARI), may hint at the mechanism whereby LUBAC promotes the formation of ubiquitin chains. They showed that HHARI, and possibly also Parkin, functions as an HECT-like E3 ligase, through a conserved cysteine residue in the second RING domain, RING2, that accepts a charged ubiquitin in a thioester intermediate before transferring the bound ubiquitin to a substrate [30]. This insight into mechanism, however, cannot explain the specific generation of linear ubiquitin linkages by HOIP, because Parkin is known to generate K48- and K63-linked chains [31,32].

Clearly we are only just beginning to explore the biochemistry of linear ubiquitin chain formation by LUBAC, and much remains to be discovered about the specificity of this complex in the exclusive generation of linear ubiquitin chains, and the exact actions of the different components within the protein complex.

## Linear ubiquitination in the TNF receptor pathway

**Figure 2:**
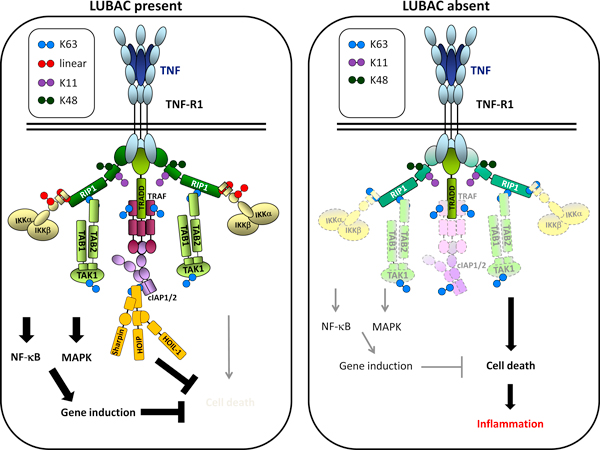
**Model of TNFR1 signaling with and without LUBAC activity**. Binding of trimeric TNF crosslinks the extracellular domains of three TNFR1 molecules and induces the formation of the TNF-RSC (also referred to as complex I). The tripartite LUBAC (ochre) is recruited to the TNF-RSC in a TRADD-, TRAF2- and cIAP-dependent manner (left panel) [16,19]. LUBAC activity in the TNF-RSC results in linear ubiquitination of RIP1 and NEMO [19] and enables the NF-κB and MAPK pathways to be activated to their full physiological extent. After a delay, and probably as a consequence of deubiquitination events at the membrane-bound TNF-RSC, the composition of the complex changes, and a second complex, complex II, appears in the cytosol [45]. Complex II (not shown) recruits FADD and caspase- 8, which are responsible for the induction of apoptosis, and includes RIP1 and RIP3, which mediate necroptosis. In the presence of LUBAC, however, the induction of cell death is prevented, probably by both stabilization of complex I by linear ubiquitination and the actions of genes induced by the NF-κB and MAPK pathways [16]. In the absence of SHARPIN (right panel), the other two LUBAC components are also drastically diminished, TNF-induced gene activation is attenuated and the TNF-RSC is destabilised, resulting in enhanced complex II formation and, consequently, cell death induction by apoptosis and necroptosis. Note that we have drawn the ubiquitin chains as diubiquitins. The actual length of the individual ubiquitin chains attached to components of the TNF-RSC - or indeed to components of any other signaling complex - is currently unknown.

Ubiquitination by K63- and K48-linked chains was already known, before the discovery of linear ubiquitin chains, to play an important part in the activation of NF-κB, arguably the most crucial output of TNFR1 signaling. Activation of the TNFR1 pathway occurs when trimeric TNF crosslinks three TNFR1 monomers to initiate formation of the TNFR1 signaling complex (TNF-RSC). As schematically illustrated in Figure 2, TNFR1 activation results in the induction of gene activation by NF-κB and mitogen-activated protein kinases (MAPKs) and, depending on the strength of these gene-activatory signals, also in cell death, which can be either apoptotic (non-inflammatory) or necroptotic (inflammatory).

NF-κB is a central transcriptional regulator in the induction of immune response genes that, in the absence of activating signals, is located in the cytoplasm. Activation of NF-κB occurs through the action of a kinase complex, referred to as the IkB kinase (IKK) complex, which consists of two catalytic subunits, IKKα (IKK1) and IKKβ (IKK2), and a critical regulatory subunit called NEMO (IKKγ). This complex is required to phosphorylate the inhibitor of NF-κB (IκB), thereby inducing its degradation and releasing NF-κB to relocate to the nucleus and bind to the promoters of immune genes. The IKK complex is recruited to the TNF-RSC through NEMO, and this results in activation of the kinase activity of this complex. MAPKs are activated as a result of recruitment of the TAB/TAK complex into the TNF-RSC. Whilst the TAB/TAK complex is currently thought to be recruited exlusively to K63-linked chains within the TNF-RSC, the IKK complex can be recruited to this complex via linear chains and, albeit with lesser affinity, also via K63- and K11-linked chains [33].

LUBAC activity was first implicated in signaling from TNFR1 when TNF-mediated NF-κB activation was shown to be impaired in primary hepatocytes from HOIL-1 knockout mice, and LUBAC was shown to form part of the signaling complex that forms on binding of TNF by the receptor, and moreover to be crucial both to the stability of the TNF-RSC and in determining the outcome of TNF signaling [16-18]. How LUBAC recruitment to the TNF-RSC influences signaling outcome is not known in detail, but it is known that NEMO, which is the regulatory component of the kinase complex that activates NF-κB, recognizes linear ubiquitin chains through its specialized ubiquitin-binding domain, UBAN (ubiquitin-binding domain present in ABINs and NEMO) [17,34]. The UBAN motif is known also to recognize ubiquitin chains with other linkages - in particular K63 chains, which are also present on components of the TNF-RSC, including on RIP1 [19]; but the UBAN of NEMO binds linear di-ubiquitin with a different topology and about 100-fold higher affinity than it does K63-linked di-ubiquitin. This suggests that the promotion of NF-κB activation by LUBAC following TNF stimulation may be due to linear ubiquitination of a component of the signaling complex whereby NEMO is recruited to, or retained in, the complex more effectively.

LUBAC also linearly ubiquitinates NEMO itself in the native TNF-RSC [19]. TNF-induced linear ubiquitination of NEMO preferentially occurs on K285 and K309, and in cells expressing a NEMO K285R/K309R mutant, NF-κB activation induced by LUBAC overexpression or by stimulation with IL-1β was reduced [18]. The mechanism of linear-ubiquitination-induced NF-κB activation has not been solved, but current data indicate that binding of NEMO to linearly linked ubiquitin induces a conformational change in the helical structure of NEMO that may promote the kinase activity of the IKK complex [17,35]. Alternatively, recognition of linear chains by NEMO conjugated to the NEMO molecules of other IKK complexes could bring the kinase domains of the respective IKK complexes into close proximity, thereby enabling trans-autophosphorylation [17], a process similar to the one that occurs between receptor tyrosine kinases when activated by ligand-induced dimerization.

Together, these findings indicate a functional role for linear ubiquitination in full gene activation by the signaling pathways triggered by TNF *in vivo*. In the absence of LUBAC components the TNF-RSC still forms and activation of NF-κB still occurs, albeit at significantly reduced levels [20,21]. Experiments with HOIP-deficient cells will be needed to strictly corroborate these findings, but it is likely that the NF-κB activation that still occurs in the absence of LUBAC is mediated by K63- and/or K11-linked chains, which are also present in the native TNF-RSC [19] and can also bind or be attached to NEMO [33,36-39].

Absence of LUBAC components also renders cells sensitive to TNF-induced cell death [16,20]. Intriguigingly, this cell death is not only apoptotic [19,20] but also necroptotic [19]. Importantly, this is also true of primary keratinocytes obtained from young, non-diseased *cpdm *mice. These mice, which are genetically deficient in SHARPIN and thus lack functional LUBAC complexes [19], have played a central part in the discovery of the physiological function of LUBAC. They present with stark immune system developmental abnormalities, and develop a chronic multi-organ inflammatory syndrome with strong manifestation in the skin (hence the name of this mutation: *chronic proliferative dermatitis *(*cpdm*)) at about 4 to 6 weeks of age [40]. The inflammatory syndrome that characterizes *cpdm *mice is apparently paradoxical, because it is generally thought that aberrantly high TNF-induced gene activation is the source of inflammation induced by this cytokine. Our finding that TNF stimulation results in aberrant death of *cpdm*-derived cells, and that this cell death has both an apoptotic and a necroptotic (and thus inflammatory) component [19,41,42], suggested a different explanation: namely, that the inflammation in *cpdm *mice could be due to inflammatory cell death consequent on the absence of SHARPIN-requiring LUBAC activity. To investigate this possibility, we crossed *cpdm *mice with TNF-deficient mice, and were able to show that even partial genetic ablation of TNF prevented the formation of inflammatory lesions in *cpdm *mice, indicating that TNF-induced cell death is indeed causative for the inflammatory phenotype that characterizes these mice [19]. It is possible that secondary necrosis, which can occur as a consequence of apoptosis, may also contribute to inflammation in *cpdm *mice.

Hence, linear ubiquitination is implicated in two different physiological processes: the development of the immune system and the prevention of chronic inflammation, where the latter effect is achieved through interference with TNF-induced cell death. Whether the aberrant cell death in the absence of LUBAC is due to reduced gene-inducing capacity of TNF, to a more direct effect of absence of linear ubiquitin chains from the signaling complexes induced by TNF, or perhaps to a combination of both these effects remains to be established. Our current suggestion for the contribution of LUBAC to these pathways is schematically illustrated in Figure 2.

## What next?

The discovery of linear ubiquitin chains and their specific ligase complex (LUBAC) has sparked considerable interest in the physiological roles of these cellular signals. Rapid progress in the delineation of protein assemblies involved in conjugation and recognition of linear ubiquitination *in vivo *have provided a platform for addressing new challenges in the field. Among them are proteomic studies of the linear ubiquitinome - the set of linearly ubiquitinated proteins in cells; analysis at atomic resolution of protein complexes implicated both in conjugation and recognition functions; and the possibility of finding novel regulatory components of LUBAC by identification of regulatory principles of LUBAC functions and of novel linear ubiquitin binding domains (LUBIDs). Interestingly, the new LUBIDs include the zinc finger (ZF) domain of HOIL-1, which has recently been shown to recognize specifically linear ubiquitin chains [43]. One of the greatest challenges, however, will be to understand how the different types of ubiquitin linkages cooperate to achieve the exact physiologically required signaling output, and how this is regulated at the level of the receptor signaling complexes. Identifying the individual ubiquitination events that occur in the TNF-RSC and determining their respective physiological roles is likely to provide valuable insight into biochemistry and function of different types of ubiquitinations, including linear ubiquitination [44].
